# Gut microbiota: a novel key player in urinary stone formation – from oxalate metabolism to systemic regulation

**DOI:** 10.1080/19490976.2026.2734634

**Published:** 2026-09-18

**Authors:** Kenan Wang, Weiwei Fan, Qizhen Tang, Quanxin Su

**Affiliations:** a Department of Urology, The First Affiliated Hospital of Dalian Medical University, Dalian, Liaoning, China

**Keywords:** Gut microbiota, gut–kidney axis, oxalate metabolism, kidney stones, probiotics

## Abstract

Kidney stones represent a globally prevalent disease with a rising incidence associated with multiple factors, including obesity and metabolic syndrome. While traditional research has primarily focused on dietary nutrition and metabolic abnormalities, recent years have seen growing recognition of the gut microbiota as a key novel regulator in stone formation. Accumulating cross-sectional evidence suggests that kidney stone patients are often associated with alterations in gut microbiota composition and function compared with healthy individuals. The gut microbiota may influence kidney stone formation risk through multiple potential mechanisms, including affecting oxalate metabolism, calcium-phosphorus absorption, short-chain fatty acid and bile acid pathways, and regulating systemic inflammatory and immune states. More importantly, this “gut–kidney axis” function of the gut microbiota holds clinical translational potential, suggesting that targeted microbial interventions may emerge as novel strategies for preventing and treating kidney stones. This review explores the association between gut microbiota and kidney stones, discussing clinical evidence, underlying mechanisms, and intervention strategies while analyzing current challenges and outlining future research directions.

Urolithiasis is a prevalent condition in urology with a reported prevalence ranging from 2% to 20% worldwide, and it primarily manifests as kidney stones. Patients often experience recurrent episodes, imposing a significant burden on healthcare systems.^1^ ^,^ ^2^ Calcium oxalate constitutes the predominant component of kidney stones (approximately 75%), followed by uric acid and magnesium ammonium phosphate (infectious stones).^3^ Stone formation results from multifactorial interactions, including genetic predisposition, dietary and hydration patterns, metabolic disorders (e.g., hyperoxaluria, hyperuricemia), and urinary tract factors (e.g., urinary stasis, infection).^4^ In recent years, metabolic factors promoting stone formation (such as obesity and type 2 diabetes) have gained increasing prominence.^5-7^ However, even after controlling traditional risk factors, stone recurrence rates remain high, suggesting the potential presence of other critical regulatory factors.

The gut microbiota, as the host's “second genome,” is closely associated with host nutrition metabolism and immune homeostasis.^8^ The concept of the “gut–kidney axis” suggests that alterations in the composition and metabolic functions of the gut microbiota may influence the urinary tract environment through multiple pathways, thereby regulating stone formation.^9^ ^,^ ^10^ As early as the late 20th century, studies identified *Oxalobacter formigenes* as capable of degrading intestinal oxalate, with its colonization deficiency potentially increasing the risk of oxalate stone disease.^11-13^ Subsequent research and clinical investigations further revealed dysbiosis in the gut microbiota of kidney stone patients, demonstrating that this impact extends far beyond a single species like *Oxalobacter formigenes*, involving alterations in the entire network of the gut microbiome. Therefore, this article reviews the latest research progress on the relationship between gut microbiota and kidney stones, focusing on the clinical evidence linking the two, potential mechanisms of action, and intervention strategies targeting the microbiota along with their clinical application prospects. It also reflects on current challenges and future research directions.

## Clinical correlation evidence between gut microbiota and kidney stones

Multiple clinical studies indicate that patients with urinary tract stones exhibit varying degrees of altered *α*-diversity in their gut microbiota (Supplementary Table 1). Overall trends indicate lower species richness and evenness in stone patients compared to healthy individuals, though variations exist across studies. Ticinesi et al.^14^ found significantly reduced Chao1 indices in the stone group (*n* = 52 recurrent idiopathic calcium stone patients vs. *n* = 48 healthy controls), while Shannon and Simpson indices showed non-significant differences, suggesting decreased species richness with relatively preserved evenness. Similar findings emerged in a U.S. pediatric cohort. Denburg et al. observed significantly reduced α-diversity in gut microbiota among 44 children with calcium oxalate stones, concurrent with fecal metabolomic abnormalities.^15^ Conversely, some small-sample studies failed to detect significant differences in α-diversity. Suryavanshi et al. found no significant difference in Shannon index between stone and control groups in patients with recurrent hyperoxaluric calculi.^16^ This inconsistency may stem from variations in sample size, dietary background, stone type, and analytical methods.

Compared to α diversity, β diversity analysis has yielded more consistent results across different studies. Nearly all population studies based on 16S rRNA or metagenomic sequencing have reported significant differences in the overall gut microbiota structure between stone patients and healthy controls. Tang et al. found significant separation of gut microbiota between stone patients and controls at the β-diversity level (ANOSIM R > 0, *p* < 0.05) through PCoA and ANOSIM analyses.^17^ Similar findings were validated in studies by Suryavanshi et al. and Ticinesi et al.^14^ ^,^ ^16^. This structural difference persists in larger cohort studies. Kim et al. observed that gut microbiota composition in individuals with past or current kidney stones significantly differed from stone-free adults, with particularly pronounced β-diversity differences in the past stone group.^18^ Metagenomic studies further corroborate this finding. Al et al. noted in a multi-site microbiome study that the gut microbiota of stone patients exhibited systemic perturbations at both compositional and network structural levels,^19^ suggesting that disruption of gut microenvironment homeostasis may be a common feature among stone-prone individuals. Overall, current evidence suggests that patients with kidney stones may show reduced or altered α-diversity in the gut microbiota, although this finding varies among studies. In contrast, β-diversity analyses more consistently indicate that the overall gut microbial community differs between stone patients and healthy individuals. These findings suggest that kidney stone disease is associated with altered microbial diversity and gut community structure, reflecting impaired gut ecosystem stability that may contribute to stone formation.

At the level of differential microbiota, although the specific genera and species reported in different studies are not entirely consistent, multiple studies have found that patients with kidney stones have relatively higher abundances of microbiota such as *Bacteroides* and *Escherichia–Shigella*, which are associated with inflammation or dysbiosis in certain cases. In contrast, healthy controls have higher abundances of *Prevotella* and certain short-chain fatty acid-producing bacteria. Stern et al. were the first to report significant enrichment of the *Bacteroides* genus in the intestines of stone patients, while *Prevotella* dominated in healthy controls.^20^ This trend was subsequently replicated in multiple studies. Tang et al. and Kim et al. both identified increased abundance of taxa such as *Bacteroides* and *Escherichia–Shigella* in stone patients, while controls showed enrichment of potentially beneficial bacteria such as *Prevotella*, *Faecalibacterium*, and *Bifidobacterium.* ^17^ ^,^ ^18^ Functionally, reductions in oxalate-degrading bacteria and short-chain fatty acid producers were particularly pronounced. Denburg et al. reported significant reductions in multiple oxalate-degrading and butyrate-producing bacteria (including *Oxalobacter formigenes*, *Bifidobacterium animalis*, and *Roseburia* spp.) among stone-forming children.^15^ Ticinesi et al. similarly observed decreased levels of butyrate-producing bacteria such as *Faecalibacterium* and *Dorea* in stone patients, which correlated negatively with 24-h urinary oxalate excretion.^14^ Furthermore, a meta-analysis incorporating 8 studies with 356 stone patients and 347 controls revealed significantly elevated relative abundances of *Bacteroides* and *Escherichia–Shigella* in patients' intestines, while *Prevotella* and other genera were more abundant in controls.^21^ This quantitatively reinforces the association between gut dysbiosis and urinary calculi.

In addition, in conditions causing gut dysbiosis and fat malabsorption, such as inflammatory bowel disease, short bowel syndrome, and post-gastrointestinal surgery, patients exhibit significantly elevated risks of developing kidney stones (primarily calcium oxalate stones).^22-25^ These clinical associations provide supportive evidence that gut dysbiosis may be linked to an increased risk of stone formation, although causality remains to be established.

## Potential multidimensional mechanisms of gut microbiota influence on kidney stones formation

### Oxalate metabolism and the gut–kidney oxalate axis

**Figure 1.**
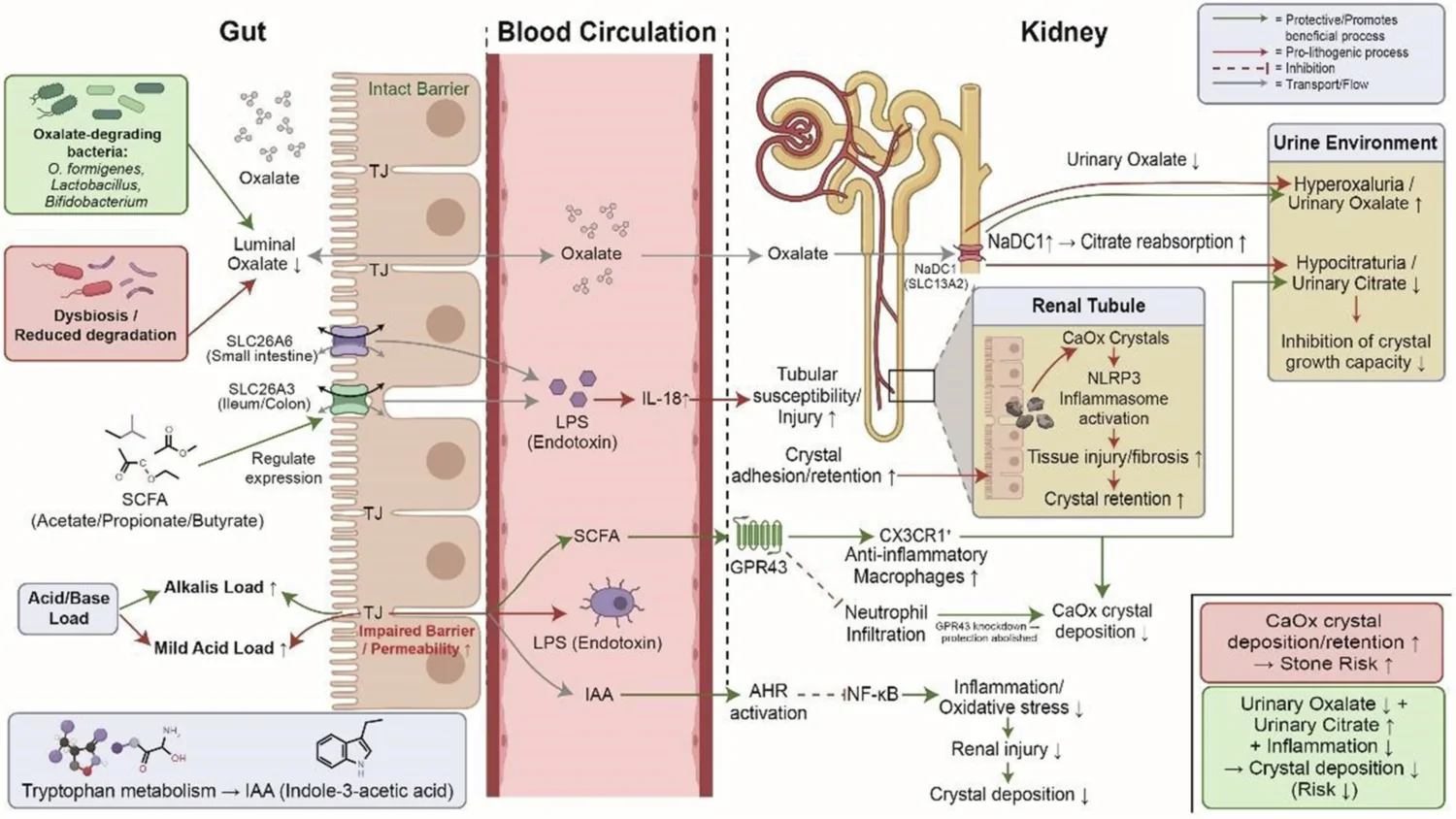
Role of gut microbiota in kidney stone.

The oxalate metabolism pathway represents the most thoroughly studied and evidence-rich mechanism within the “gut microbiota–metabolites–stone” axis. Disrupted oxalate metabolism is a key promoter of calcium oxalate stone formation. Under normal conditions, the intestinal absorption rate of dietary oxalate is relatively low (approximately 10%–15%), with a significant portion of oxalate being degraded and utilized by gut bacteria.^26^ Gut microbiota are thought to play an important role in host oxalate metabolism. On the one hand, *Oxalobacter formigenes* can use oxalate as a carbon source and break it down into metabolites such as formic acid.^11^ Symbiotic bacteria, such as *Lactobacillus* and *Bifidobacterium*, also possess the ability to degrade oxalate,^27^ ^,^ ^28^ thereby reducing intestinal oxalate absorption. On the other hand, oxalate transporters expressed on the intestinal epithelium (e.g., SLC26A6, SLC26A3) can secrete oxalate back into the gut or absorb it into the body.^9^ ^,^ ^29^ Among these, the oxalate transporter SLC26A6 is primarily expressed in the small intestine, while SLC26A3 is highly expressed in the ileum, cecum, and colon.^30^ ^,^ ^31^ Multiple animal studies demonstrate that SLC26A6 knockout mice exhibit enhanced net oxalate absorption in the ileum, leading to renal crystal deposition, hyperkalemia, and hyperoxaluria.^32-35^ SLC26A3 knockout mice also show a 66% reduction in 24-h urinary oxalate levels compared to controls.^36^ These studies support a mechanistic link between oxalate transporters, oxalate metabolism, and renal CaOx crystal deposition. Microbial metabolites can also influence these transport processes. Liu et al. found that gut metabolites including acetate, propionate, and butyrate reduce renal CaOx crystal and urinary oxalate levels in rats by modulating oxalate transporter expression.^37^ In summary, within the “gut–kidney–oxalate” axis, gut microbiota and their metabolites may facilitate intestinal oxalate degradation and reduce oxalate absorption, thereby potentially lowering renal oxalate burden and lowering urinary oxalate concentration, thereby inhibiting calcium oxalate crystal formation. Conversely, dysbiosis impairing oxalate degradation may lead to increased dietary oxalate absorption, resulting in hyperoxaluria and increasing the risk of calcium oxalate crystallization or stone formation (Figure 1).

### Gut microbiota and the absorption and excretion of trace minerals

In addition to oxalate, kidney stone formation is also influenced by urinary concentrations of ions such as calcium, phosphate, and magnesium, which affect urinary supersaturation and crystal formation.^4^ Gut microbiota may similarly play a role in stone formation by affecting the host's absorption and metabolism of these minerals. Joris Vrielinck et al. reported case analyses of two outbreaks of urolithiasis in pig farms, revealing disrupted calcium and phosphorus metabolism in the intestines and diets of affected herds. They hypothesized that gut microbiota may cause abnormal urinary ion concentrations by influencing calcium and phosphorus absorption rates and systemic homeostasis.^38^ In humans, short-chain fatty acids (SCFAs), particularly acetate and propionate, have been shown to enhance calcium absorption in the distal colon and rectum, suggesting that microbial metabolites may influence intestinal calcium handling and, indirectly, urinary calcium excretion.^39^ ^,^ ^40^ However, the relationship among SCFAs, luminal calcium, oxalate absorption, and urinary stone risk is complex. The classical mechanism of enteric hyperoxaluria occurs in conditions of fat malabsorption, where unabsorbed fatty acids bind luminal calcium, leaving less free calcium available to complex with oxalate. As a result, more soluble oxalate remains available for intestinal absorption, leading to increased urinary oxalate excretion and a higher risk of calcium oxalate crystallization.^41^ In contrast, if reduced intestinal calcium absorption simply leaves more free calcium in the gut lumen, this would theoretically be expected to bind oxalate and reduce, rather than increase, oxalate absorption. Therefore, whether reduced SCFA production directly increases urinary oxalate remains speculative and likely depends on luminal calcium availability, fat absorption status, bile acid metabolism, intestinal permeability, and oxalate transporter activity. Separately, gut dysbiosis may influence urinary calcium excretion through effects on intestinal calcium absorption, vitamin D-related endocrine pathways, parathyroid hormone regulation, and low-grade inflammation.^42^ ^,^ ^43^ In some contexts, these pathways may contribute to hypercalciuria and increased calcium salt supersaturation. Clinical studies have shown that kidney stone formers often exhibit abnormalities in the bone–mineral metabolism axis, including reduced bone mineral density and hypercalciuria.^44-47^ However, the extent to which gut microbiota mediate these associations requires further investigation.

Notably, healthy microbiota are also crucial for maintaining stone-inhibiting factors in urine. In healthy individuals, urinary citrate chelates calcium ions and prevents crystal growth.^48^ However, individuals with stones often exhibit hypocitraturia, leading to reduced anti-stone capacity.^49^ ^,^ ^50^ SCFAs (e.g., acetate) produced by gut microbiota, when absorbed and metabolized, act as “metabolizable anions” to generate an alkaline load in the body, thereby producing a blood/body fluid alkalinizing effect.^51^ Systemic alkaline load or urinary alkalization reduces proximal tubular reabsorption of citrate and increases urinary citrate excretion. Clinically, alkalinizing therapies (e.g., bicarbonate loading) elevate urinary pH and significantly increase urinary citrate levels.^48^ ^,^ ^52^ Conversely, under mild acid load or acid retention conditions, expression of the proximal tubular citrate transporter NaDC1 (SLC13A2) may be upregulated, promoting increased citrate reabsorption and resulting in decreased urinary citrate.^53^ ^,^ ^54^ Therefore, when the abundance of short-chain fatty acid-producing bacteria decreases, the human body may be more prone to mild acid load and reduced urinary citrate levels. This weakens citrate's inhibitory effect on calcium salt crystallization, hindering stone prevention.^48^ Chen et al. found significantly reduced SCFA-producing bacteria in stool samples from stone patients, with this dysbiosis closely associated with decreased fecal and plasma SCFA levels and reduced 24-h urinary citrate.^55^ Zhang et al. also observed a significantly higher incidence of hypocitraturia in recurrent stone patients,^56^ suggesting gut dysbiosis as a potential contributing factor.

In summary, normal microbiota may prevent crystal deposition by maintaining trace mineral absorption and urinary inhibitory factor levels. However, the precise mechanisms underlying gut microbiota-mineral metabolism remain complex and warrant further investigation.

### Gut barrier-immune inflammatory response

Increasing evidence indicates that stone formation is not merely a physicochemical crystallization process; the host's immune inflammatory state and epithelial barrier function also profoundly influence crystal adhesion, growth, and clearance. Therefore, the “intestinal barrier integrity–immune inflammation axis” constitutes another mechanism through which the microbiota influences stone formation. A healthy intestinal mucosal barrier prevents bacteria and their metabolites (e.g., endotoxins) from entering the bloodstream.^57^ ^,^ ^58^ Dysbiosis often weakens this barrier function, leading to endotoxin translocation and elevated pro-inflammatory cytokine levels. A genetic study employing two-sample Mendelian randomization analysis revealed that alterations in the abundance of certain gut microbiota may influence kidney stone development by regulating specific pro-inflammatory factors such as interleukin-18 (IL-18).^59^ Elevated abundance of *Actinobacteria* phylum and *Rhodospirillales* order microbiota was associated with reduced kidney stones, with IL-18 partially mediating this protective effect. Hou et al. reported a causal association between genetic abundance of *Bacteroides* and *Prevotella* and kidney stones, identifying CD28 expression on CD39^+^CD4^+^ T cells as a mediator in the *Prevotella-to-KSD* protective pathway.^60^ These studies suggest the gut microbiota-immune axis may influence renal stone susceptibility via cytokine signaling.

A chronic inflammatory environment can alter the renal microenvironment, such as by upregulating expression of adhesion molecules and chemokines in renal tubular cells, facilitating crystal adhesion to damaged renal papillae and crystal accumulation.^61^ The kidney's immune response to crystals is also a critical determinant of stone formation. Multiple studies indicate that calcium oxalate crystals induce local renal inflammation, activate NLRP3 inflammasomes, and recruit macrophages, leading to tissue injury and fibrosis that further promotes long-term crystal retention and growth.^62-64^ Jin et al. found that supplementing oxalate-stone model mice with SCFAs like butyrate and propionate, via their receptor GPR43, increased anti-inflammatory phenotype macrophages (CX3CR1⁺ macrophages) and reduced pro-inflammatory neutrophil infiltration, significantly decreasing renal calcium oxalate crystal deposition.^65^ When GPR43 was genetically knocked down in mouse kidneys, this protective effect of SCFAs disappeared, demonstrating that SCFAs protect the host from stone damage via immune pathways. Furthermore, Jing et al. discovered that certain tryptophan metabolites derived from gut bacteria exhibit anti-inflammatory effects. Indole-3-acetic acid (IAA), a small molecule produced by gut symbionts metabolizing tryptophan, activates the host aryl hydrocarbon receptor (AHR), thereby inhibiting NF-κB-mediated inflammatory pathways.^66^ In rat models, IAA administration alleviated oxidative stress and inflammatory injury in kidneys induced by calcium oxalate crystals, significantly reducing crystal deposition. This suggests gut microbiota metabolites may influence stone formation and tissue responses through immune regulation.

Collectively, chronic inflammation induced by gut microbiota dysbiosis is considered a potential mechanism promoting stone formation. The “microbiota-barrier-inflammation-stone” axis links microbial function to host immunity and the microenvironment. Gut microbiota may influence stone outcomes by maintaining the barrier and supplying anti-inflammatory metabolites to modulate host immune responses.

### Bile acid and purine metabolism

The reabsorption and degradation of bile acids in the intestine are highly dependent on gut symbionts.^67^ ^,^ ^68^ When ileal malabsorption or dysbiosis leads to bile salt retention in the colon, it can cause fat and calcium binding, resulting in increased oxalate absorption and inducing hyperoxaluria as previously described.^41^ Consequently, patients with small bowel resection or bile acid malabsorption syndrome are prone to developing calcium oxalate stones. Disrupted gut microbiota-mediated bile acid metabolism may play a role in this process. However, direct evidence linking bile acids, microbiota, and stone formation remains limited, necessitating further metabolomics research for clarification.

In purine metabolism, uric acid stone formation is associated with hyperuricosuria and an acidic urinary environment. Gut microbiota play an auxiliary role in purine metabolism and uric acid clearance, with approximately one-third of uric acid being degraded by gut bacteria. For instance, *Akkermansia* and certain *Bacillota* possess uricase enzymes capable of breaking down uric acid.^69^ ^,^ ^70^ Dysbiosis may reduce the efficiency of alternative intestinal uric acid excretion pathways, promoting uric acid retention and increasing the risk of stone formation.^71^ Furthermore, intermediates in purine metabolism (e.g., hypoxanthine, xanthine) may also be modulated by microbiota. Liu et al. identified widespread presence of uric acid degradation gene clusters in gut bacteria, which metabolize uric acid into xanthine or SCFAs. Weakened anaerobic microbiota may increase hyperuricemia risk.^72^ Therefore, it can be inferred that gut microbiota indirectly influence the formation of calcium oxalate and uric acid stones through disrupted bile acid and uric acid metabolic pathways. However, these mechanisms have been relatively understudied to date and warrant further exploration.

## Targeted gut microbiota intervention strategies and clinical applications

### Probiotics and synbiotics

The therapeutic efficacy of probiotics for stones remains controversial. In an early open-label trial, Campieri et al. administered high-dose probiotic mixtures orally for 4 weeks to 6 patients with calcium oxalate stones and mild hyperoxaluria. Results showed a reduction in 24-h urinary oxalate from approximately 55.5 mg at baseline to 33.5 mg, with sustained low levels maintained for 1 month post-treatment discontinuation (*p* < 0.05).^73^ However, Goldfarb et al. reported differing results. In their randomized double-blind controlled trial, no significant effect of a four-strain lactic acid bacteria mixture (Oxadrop) on urinary oxalate was observed in 20 subjects with idiopathic hyperoxaluria.^74^ Similarly, Lieske et al. RCT showed that under strict low-oxalate dietary control, adding probiotics or synbiotics did not further reduce urinary oxalate excretion or calcium oxalate supersaturation in patients.^75^ Furthermore, both Siener et al. trial inducing dietary hyperoxaluria in healthy volunteers and Tavasoli et al. placebo-controlled trial (initially enrolling 100 subjects, with 64 completing) yielded negative results, indicating no difference in urinary oxalate reduction between the probiotic group and the control group.^76^ ^,^ ^77^ Further stool cultures in the latter study revealed that the ingested probiotics were undetectable in the feces of most subjects, suggesting that failure of intestinal colonization may explain the lack of significant efficacy. Therefore, some scholars have proposed adopting the concept of combined microbiota supplementation rather than single-strain supplementation to rebuild the intestinal oxalate metabolic network. Miller et al. found in a clinical microbiome study that healthy individuals harbored a broader oxalate-associated bacterial network, rather than relying solely on a single oxalate-degrading species.^78^

Several newly screened probiotic strains have also demonstrated promising effects in animal models. *Lactobacillus plantarum AR1089*, isolated by Xu et al., degraded approximately 20% of oxalate *in vitro*. When administered orally to rats on a high-oxalate diet, it reduced serum creatinine and blood urea nitrogen levels, alleviated renal fibrosis and calcium oxalate deposition, and downregulated expression of renal tissue injury and inflammatory markers.^79^ Additionally, Wei et al. used *Lactobacillus paracasei LPN1* to treat rats with calcium oxalate stones, finding it increased tight junction protein expression in the intestinal epithelium and reduced endotoxin translocation, thereby mitigating renal injury and urolith formation.^80^ These studies indicate that optimizing strain properties, such as combining oxalate reduction with anti-inflammatory benefits, is key to enhancing probiotic efficacy. Beyond probiotics, synbiotics (probiotic + prebiotic combinations) represent another noteworthy strategy. Prebiotics, typically oligosaccharides or dietary fibers metabolized by beneficial gut bacteria, promote the survival and function of colonized microbes. An animal study demonstrated that a synbiotic formulation combining oxalate-degrading bacteria with galacto-oligosaccharides effectively restored the abundance balance of anti-oxalate microbiota and prevented calcium oxalate stone formation.^81^ Synbiotics hold promise in overcoming the limitations of poor colonization by probiotics alone, offering more stable effects for microbiota regulations.

### Fecal microbiota transplantation (FMT)

As a method for reshaping the gut microbiota, fecal microbiota transplantation remains in the exploratory phase for kidney stones. Animal model studies provide preliminary evidence: Stern et al. transplanted fecal microbiota from healthy donors (Zucker lean rats) into germ-free mice, observing approximately 55% reduced calcium excretion and 24% reduced oxalate in recipient mice's 24-h urine.^82^ These findings suggest that comprehensive microbiota reconstruction may improve host oxalate metabolism and urinary physicochemical conditions, potentially reducing stone risk factors. Conversely, transplanting dysbiotic microbiota from kidney stone patients into germ-free or antibiotic-pretreated animals replicates a stone-prone phenotype. In Hunthai et al. study, transplanting gut microbiota from 8 calcium oxalate stone patients into rats induced recipient rats to exhibit urinary disorders similar to patients, including high urinary calcium, high urinary oxalate, and high supersaturation, accompanied by impaired intestinal barrier function (downregulation of tight junction protein ZO-1) and increased expression of oxalate transporter SLC26A6.^83^ Although no significant renal crystallization occurred within 4 weeks, these alterations indicate that stone-associated dysbiosis may promote stone risk factors by increasing intestinal permeability and oxalate absorption. Conversely, recent studies demonstrate the potential of FMT to prevent stone formation in animal models. An et al. transplanted microbiota from healthy humans into rats with hyperoxaluria induced by a high-oxalate diet.^84^ Results showed improved renal calcium oxalate crystal deposition and renal injury in rats, alongside reversal of gut dysbiosis and restoration of key probiotic genera (e.g., *Allobaculum*) abundance. FMT also repaired the intestinal mucosal barrier disruption caused by high oxalate intake, thereby reducing oxalate crystal formation. This animal evidence collectively supports the biological rationale for FMT as an intervention strategy. However, clinical trial data remain lacking, necessitating further research on its safety and feasibility.

### Live microorganism biotherapy and engineered microbial strategies

Beyond traditional probiotics, emerging approaches are testing specific functional strains as “living biotherapeutics.” The most representative example is the formulation application of *Oxalobacter formigenes*, an oxalate-degrading bacterium. *Oxalobacter* is an intestinal bacterium that specifically uses oxalate as a substrate. Multiple observational studies indicate lower detection rates of this bacterium in the intestines of stone patients.^11^ ^,^ ^12^ Based on this, some researchers have attempted to reduce host oxalate burden through *Oxalobacter* supplementation. Hoppe et al. conducted clinical trials for primary hyperoxaluria in 2011 and 2017.^85^ After 8 weeks of daily oral administration of freeze-dried *Oxalobacter* to pediatric subjects, no significant difference in 24-h urinary oxalate levels was observed between the treatment group and the placebo group. Although fecal testing confirmed successful *Oxalobacter* colonization and good safety, the primary efficacy endpoint was not met. The recent ePHex Phase III trial extended Oxabact treatment to 52 weeks to assess efficacy. Results showed a slight decrease in plasma oxalate levels from baseline in the treatment group, while the placebo group showed an increase. However, the difference between groups remained non-statistically significant at one-year follow-up (*p* = 0.064).^86^ Overall, controlled clinical evidence for *O. formigenes* supplementation in primary hyperoxaluria remains limited, with several randomized trials failing to meet their primary oxalate-lowering endpoints.

These negative or inconclusive findings may not indicate a complete lack of biological potential, but rather suggest that the efficacy of *O. formigenes* is likely influenced by multiple factors, including colonization persistence, formulation viability, strain-specific properties, host disease severity and antibiotic exposure. Therefore, more recent studies have begun to refine patient selection by showing that the response to *O. formigenes* supplementation may depend on the baseline functional capacity of the resident gut microbiota. Fargue et al. reported that live *O. formigenes* administration in initially non-colonized healthy adults was safe, achieved sustained intestinal colonization, and reduced urinary oxalate excretion.^87^ Subsequent analysis further showed that individuals with lower baseline abundance of oxalate-degrading bacteria and genes, particularly *oxc* and *frc*, exhibited greater urinary oxalate reduction after *O. formigenes* colonization.^88^ These findings may help explain the inconsistent results of earlier trials and suggest that *O. formigenes*-based therapy may be most effective in selected individuals with low baseline oxalate-degrading capacity.^89^

An alternative strategy focuses on engineered strains. Synthetic biology-based modification of gut bacteria to enhance oxalate reduction represents a frontier exploration, exemplified by Synlogic's engineered *Escherichia coli* Nissle (strain SYNB8802). Kurtz et al. reported that SYNB8802 significantly reduced urinary oxalate excretion in mouse and non-human primate models. *In vitro* and *in vivo* data modeling predicted clinically meaningful urinary oxalate reduction in humans.^90^ This novel “synthetic probiotic” is viewed as a promising treatment for enterogenic hyperoxaluria. Overall, live microbial therapeutics and engineered bacteria strategies offer innovative pathways for gut microbiota intervention. However, their clinical translation faces multiple challenges, including the need for efficacy validation, regulatory approval, and patient acceptance.

## Current challenges and future research directions

Despite rapid advances in gut microbiota-kidney stone research, critical bottlenecks persist. First, most of the existing evidence comes from cross-sectional or correlational studies, making it difficult to determine whether gut microbiota dysbiosis is a cause, a consequence, or a bidirectional factor that interacts with host metabolic abnormalities in the development of kidney stones. At the same time, the composition of the gut microbiota itself is susceptible to various confounding factors, including dietary patterns, dietary oxalate and calcium intake, obesity, diabetes and other metabolic diseases, geographic and ethnic differences, drug exposure, probiotic use, and antibiotic use. Antibiotic therapy and dietary interventions administered to patients with kidney stones during diagnosis and treatment may further alter the gut microbiota, thereby increasing the complexity of interpreting the results. Therefore, future studies need to establish more rigorously designed prospective longitudinal cohorts, systematically collect and adjust for the aforementioned clinical and lifestyle variables, and dynamically track the temporal relationship between changes in the microbiota and the onset or recurrence of kidney stones. Concurrently, animal models and *in vitro* experiments are needed to advance from “correlation” to “causation” at the mechanistic level. While the oxalate metabolic pathway is relatively well-understood, the molecular mechanisms by which microbiota influence calcium-phosphorus metabolism, immune inflammation, and crystal adhesion/growth remain unclear. Key areas warranting further exploration include the signaling pathways through which specific microbiota or metabolites act on the kidneys, and the role of inflammatory mediators such as IL-18 in these processes. Second, microbiota exhibit high individual variability and complex ecological interactions. Probiotic colonization is influenced by preexisting community homeostasis and competitive exclusion, while FMT involves donor-recipient matching and post-transplant evolutionary uncertainty. Future approaches may involve precision interventions through dynamic monitoring and feedback regulation, exploring combination therapies and “niche occupation” strategies to enhance microbial colonization and therapeutic efficacy. Finally, safety and ethical concerns cannot be overlooked. Probiotics may cause infections or transmit drug-resistant genes in immunocompromised individuals, and FMT carries risks of pathogen transmission. Future efforts should establish long-term follow-up and monitoring systems while evaluating efficacy, advancing the translational application of microbiome therapies under controlled conditions.

The gut microbiota, as a key factor influencing host metabolism and immunity, has been demonstrated to be closely associated with the development of kidney stones. Patients with stones commonly exhibit dysbiosis, including a lack of oxalate-degrading bacteria, reduced short-chain fatty acid production, and proliferation of potential pathogens. These alterations may contribute to stone risk through the “gut–kidney axis” at multiple levels, although causal relationships remain to be fully established. Modulating gut microbiota is emerging as a novel therapeutic approach for kidney stone prevention and treatment. Although current microbiota interventions remain in the exploratory phase clinically, deepening understanding of the microbiota-stone mechanism holds promise for developing more effective and safer microbiological therapies, opening new chapters in stone diagnosis and treatment.

## Supplementary Material

Supplementary Table 1.docx

## Data Availability

Not applicable.
