# Supplementary material for: Gut microbiota: a novel key player in urinary stone formation – from oxalate metabolism to systemic regulation
Source: Gut Microbes. 2026 Sep 18;18(1):2734634. doi: 10.1080/19490976.2026.2734634 (PMC13596874; doi:10.1080/19490976.2026.2734634)
Supplement: Supplementary file 1 [file KGMI_A_2734634_SM6743.docx]

Supplementary Table 1. Characteristics of studies on the association between Gut Microbiota and Kidney Stones

| Author/Country/Year | Population/Sample size | Method | α diversity differences | β diversity differences | Taxa with higher abundance in kidney stone group | Taxa with higher abundance in control group |
| --- | --- | --- | --- | --- | --- | --- |
| Stern et al. / USA / 2016 | Kidney stones 23; controls 6 | 16S | NR | NR | Bacteroides | Prevotella |
| Suryavanshi et al. / India / 2016 | Kidney stones 24; controls 15 | 16S (with targeted frc detection) | Chao1, observed species, PD, Shannon, Simpson: no difference | UniFrac PCoA: compositional differences between groups | Firmicutes、Proteobacteria、TM7 | Prevotella copri、Dialister |
| Ticinesi et al. / Italy / 2018 | Kidney stones 52 vs controls 48; metagenomic subset 17 vs 17 | 16S; subset metagenomics | Chao1: decreased in kidney stone group (p=0.024); Shannon and Pielou: no difference | Weighted UniFrac and Bray–Curtis: significant differences | NR | Faecalibacterium、Enterobacter、Dorea |
| Tang et al. / China / 2018 | Kidney stones 13; controls 13 | 16S | Observed, ACE, Chao1, Shannon, Simpson: no difference | NMDS separation; ANOSIM/MRPP: significant differences | Proteobacteria、Actinobacteria；Pseudomonadales、Erysipelotrichales；Moraxellaceae、Erysipelotrichaceae | Bacteroidetes、Firmicutes、Fusobacteria |
| Zampini et al. / USA / 2019 | Controls 43; kidney stones 24 | Multilocus 16S + urine metabolomics | NR | No difference | Lachnospiraceae | NR |
| Denburg et al. / USA / 2020 | Children: kidney stones (CaOx) 44 vs controls 44 | Metagenomics + metabolomics | Richness (P<0.001) and Shannon (P=0.01): decreased in kidney stone group | NR | NR | Eubacterium faecalis/ faecium、Bifidobacterium animalis、Lactobacillus spp.、Roseburia spp. |
| Chen et al. / China / 2021 | Overall: kidney stones 57 vs controls 51; sequencing subset: 30 vs 30 | 16S | Chao1, Shannon, Simpson: no difference | PCA: significant difference | Bacteroidetes；Bacteroides、Phascolarctobacterium | Firmicutes、Verrucomicrobia；Akkermansia、Faecalibacterium、Lactobacillus |
| Zhao et al. / Country NR / 2021 | Kidney stones 91; controls 75 | 16S | NR | NR | Bacteroides、Prevotella_9 | Blautia、Bifidobacterium；Lachnoclostridium |
| Deng et al. / China / 2022 | 20 upper urinary tract stone patients, paired pre- and post-surgery | 16S | ACE and Simpson: no difference | NR | Preoperative: increased Pseudomonadaceae, Pseudomonadales, and Pseudomonas | Postoperative: increased Enterobacteriales, Enterobacteriaceae, Gammaproteobacteria, and Escherichia |
| Kim H-N et al. / South Korea / 2022 | Cohort (n=915): G0 no stones 732; G1 incident 97; G2 history/current 86 | 16S (V3–V4, MiSeq) | Evenness: lower in G2; Observed, Faith PD, and Shannon: no difference | NR | Phascolarctobacterium、Fusobacterium | Bifidobacterium、Faecalibacterium、Dorea、Ruminococcus、Blautia、Coprococcus、Dialister、Lachnospira、Eubacterium 等 |
| Yuan et al. / China / 2022 | kidney stones 117; controls 105 | 16S | NR | β: significant compositional differences between groups | NR | NR |
| Al et al. / Canada / 2023 | Kidney stones 83 vs controls 30; fecal metagenomics: controls 25/kidney stones 36 | Fecal metagenomics | Shannon and Evenness: decreased in kidney stone group | PCA: significant difference (P=0.0054) | Eggerthella lenta、Flavonifractor plautii、Ruminococcus obeum 等 | Fusicatenibacter saccharivorans, etc. |
| Hussain et al. / Taiwan, China / 2024 | Three sample types: healthy feces/patient feces/stone samples (n per group: NR) | PacBio sequencing targeting the full-length 16S rRNA gene | Patient fecal community structure was weaker than that of healthy feces (specific α/β metrics NR) | NR | Common in stone samples: Morganella morganii, Ureaplasma parvum, etc. | Enriched in healthy feces: Bacteroides vulgatus, Prevotella plebeius, Faecalibacterium prausnitzii, etc. |

Abbreviations: PCoA Principal coordinate analysis, NMDS Non-metric multi-dimensional scaling, PCA Principal component analysis
